# Pregnancy as a Stress Test for β-Cell Autoimmunity: The Autoimmune Phenotype of Gestational Diabetes Mellitus

**DOI:** 10.3390/ijms27219896

**Published:** 2026-11-04

**Authors:** Vesselina Yanachkova, Velizar Shivarov, Nikolay Kostadinov, Zlatko Kirovakov, Angel Yordanov

**Affiliations:** 1 Research Institute, Medical University-Pleven, 5800 Pleven, Bulgaria; 2 Department of Endocrinology, Specialized Hospital for Active Treatment of Obstetrics and Gynaecology “Dr Shterev”, 1330 Sofia, Bulgaria; 3 Department of Clinical Hematology, St. Sophia General Hospital, 1618 Sofia, Bulgaria; 4 Department of Experimental Research, Medical University-Pleven, 5800 Pleven, Bulgaria; 5 Faculty of Public Health and Health Care, Burgas State University, 8000 Burgas, Bulgaria; n.kostadinov_m.d@abv.bg (N.K.); kirovakov@yahoo.com (Z.K.); 6 Department of Gynecologic Oncology, Medical University-Pleven, 5800 Pleven, Bulgaria; angel.jordanov@gmail.com

**Keywords:** gestational diabetes mellitus, autoimmune phenotype, islet autoantibodies, GADA, β-cell dysfunction, C-peptide, biomarkers, precision medicine

## Abstract

Gestational diabetes mellitus (GDM) is increasingly recognized as a heterogeneous metabolic disorder comprising pathophysiological phenotypes that differ in their underlying mechanisms, clinical presentation and long-term outcomes. While pregnancy-induced insulin resistance remains the predominant pathogenic mechanism, a subset of women develops hyperglycemia largely because of reduced pancreatic β-cell reserve accompanied by islet autoimmunity. This autoimmune phenotype links gestational diabetes with autoimmune diabetes, but its boundaries and clinical significance remain incompletely defined. We aimed to review current knowledge of the autoimmune phenotype of GDM, with focus on immune pathophysiological mechanisms, biomarkers, clinical characteristics and implications for precision medicine, and to distinguish findings demonstrated directly in GDM from mechanisms extrapolated from type 1 diabetes (T1D). This narrative review was based on a structured PubMed/MEDLINE search from database inception through the end of October 2026, supplemented by targeted searches, manual screening of reference lists, and relevant clinical guidelines and consensus documents. Evidence obtained directly in women with GDM was considered alongside selected mechanistic literature from T1D and adult autoimmune diabetes. In some women with GDM, islet autoantibodies are associated with impaired β-cell function and an increased risk of autoimmune diabetes after delivery. The cellular mechanisms underlying these associations remain insufficiently studied in GDM. Pregnancy may act as a physiological metabolic stress test that exposes previously compensated β-cell dysfunction associated with islet autoimmunity, although its influence on the underlying autoimmune process remains uncertain. Islet autoantibodies are enriched among women with GDM, but their frequency varies widely between populations and assays and is lower after delivery than during pregnancy. The number and persistence of antibodies, particularly when combined with reduced endogenous insulin secretion, carry considerably more prognostic weight than a single positive result. Cellular immune mechanisms are well characterized in T1D but remain largely unvalidated in GDM. Recognition of the autoimmune phenotype extends the concept of GDM beyond a purely glucose-based classification. It is best viewed as a spectrum ranging from isolated seropositivity to pregnancy-unmasked autoimmune diabetes rather than as a uniform independent entity. A phenotype-oriented approach combining clinical features, autoantibody profiles and measures of β-cell function may facilitate earlier recognition of women at risk and more individualized follow-up, but universal autoantibody screening and antibody-specific treatment are not currently supported.

## 1. Introduction

Gestational diabetes mellitus (GDM) is the most common metabolic disorder of pregnancy and is associated with adverse outcomes for both mother and fetus. It is defined as hyperglycemia first recognized during pregnancy that does not meet the criteria for overt pre-existing diabetes. Resolution after delivery is common but is not part of the definition, and persistent dysglycemia postpartum requires reclassification [1,2,3,4,5,6]. This definition, however, provides a simplified view of the condition. GDM is better regarded as a heterogeneous syndrome that encompasses several biological phenotypes, each with different pathogenetic mechanisms, metabolic characteristics and clinical manifestations [7,8]. Women with GDM differ considerably in insulin sensitivity, endogenous insulin secretion, genetic predisposition, inflammatory activity and degree of pancreatic β-cell dysfunction. On the basis of these differences, insulin-resistant, insulin-deficient, mixed and autoimmune phenotypes have been described. These subgroups are not isolated categories but exist on a biological continuum in which several mechanisms often coexist, while one usually predominates clinically. The autoimmune phenotype is of particular interest. In this subgroup, hyperglycemia results not only from pregnancy-induced insulin resistance but also from reduced β-cell reserve associated with islet autoimmunity. Pregnancy markedly increases insulin requirements, and previously compensated defects in insulin secretion may therefore become clinically apparent. For this reason, pregnancy has been described as a physiological metabolic stress test capable of revealing latent autoimmune β-cell dysfunction in genetically predisposed women [9,10].

The autoimmune phenotype shares several biological features with type 1 diabetes (T1D). It is characterized by circulating autoantibodies against islet cells (ICA), glutamic acid decarboxylase (GADA), insulinoma-associated antigen-2 (IA-2A), zinc transporter 8 (ZnT8A) and insulin (IAA) [11,12]. These autoantibodies are markers of islet-directed autoimmunity and provide important information about the mechanisms underlying hyperglycemia in pregnancy. Advances in immunology have suggested that alterations in T-cell subsets, low-grade inflammation, cytokine-mediated β-cell injury and HLA-related genetic susceptibility contribute to autoimmune diabetes. Longitudinal studies have shown that some antibody-positive women with GDM develop T1D or a slower form of autoimmune diabetes after delivery [9,10,13,14,15], the autoimmune pathogenesis of GDM has been reviewed [16], and a recent meta-analysis has pooled the prevalence of islet autoantibodies in GDM [17].

Recent publications have reviewed the proposed autoimmune pathogenesis of GDM and progression to T1D [16], and synthesized evidence on islet-autoantibody prevalence and postpartum diabetes risk [17]. Building on these contributions, the present review examines pregnancy as a metabolic challenge to β-cell functional reserve and considers how islet autoimmunity should be interpreted within this framework. Its emphasis is on the relationship between autoantibody status, functional impairment and the clinical course, while distinguishing metabolic unmasking from possible modification of the autoimmune process. Isolated seropositivity, an autoimmune phenotype accompanied by impaired β-cell function, and autoimmune diabetes first recognized during pregnancy are therefore considered separately. Evidence obtained directly in women with GDM is explicitly distinguished from mechanisms extrapolated from T1D and adult autoimmune diabetes (Table 1). This integrated interpretation provides the basis for the proposed approach to selective evaluation and postpartum follow-up, while identifying the evidence needed to validate it.

## 2. Methods

### 2.1. Review Approach

We conducted a narrative review of islet autoimmunity in gestational diabetes mellitus (GDM), with emphasis on autoantibody findings, β-cell function, possible immunological mechanisms, and their clinical relevance during and after pregnancy. The structure and reporting of the review were informed by the SANRA recommendations for narrative reviews [18]. As evidence specific to autoimmune manifestations of GDM remains limited, studies performed directly in women with GDM were considered alongside selected literature from type 1 diabetes (T1D) and adult autoimmune diabetes. Findings derived from these broader populations were used only to support the discussion of mechanisms or concepts that have not been sufficiently studied in GDM. No meta-analysis or other quantitative synthesis was undertaken.

### 2.2. Literature Search

The review covers literature published through the end of October 2026. PubMed/MEDLINE was searched using a combination of MeSH terms and title/abstract keywords for gestational diabetes, islet autoimmunity and diabetes-associated autoantibodies. The documented main search strategy was (“Diabetes, Gestational” [Mesh] OR “gestational diabetes” [tiab] OR “gestational diabetes mellitus” [tiab] OR GDM [tiab]) AND (“Autoantibodies” [Mesh] OR “Autoimmunity” [Mesh] OR autoimmun*[tiab] OR autoantibod*[tiab] OR “islet antibodies” [tiab] OR “islet cell antibodies” [tiab] OR “islet-cell antibodies” [tiab] OR GADA [tiab] OR GAD65 [tiab] OR “glutamic acid decarboxylase antibodies” [tiab] OR “glutamic acid decarboxylase autoantibodies” [tiab] OR IA-2A [tiab] OR “IA-2 antibodies” [tiab] OR “IA-2 autoantibodies” [tiab] OR ZnT8A [tiab] OR “zinc transporter 8 antibodies” [tiab] OR “zinc transporter 8 autoantibodies” [tiab] OR “insulin antibodies” [tiab] OR “insulin autoantibodies” [tiab]). The main search retrieved 473 unique records. No language or publication-type filters were applied at retrieval; only English-language publications were considered for inclusion. Additional topic-specific searches addressed C-peptide and β-cell function, postpartum development of autoimmune diabetes, HLA susceptibility, immune mechanisms, autoantibody assays and emerging biomarkers. Reference lists of relevant primary studies and reviews were also screened. Clinical recommendations were informed by relevant guidelines and consensus documents concerning GDM, autoimmune diabetes and follow-up after pregnancy. The narrative synthesis cites 44 sources overall, including primary studies, reviews, methodological publications and clinical guidelines. Fourteen of these cited sources were also retrieved by the documented main search. The remaining cited sources provide additional clinical, mechanistic, methodological or contextual support. The total number of cited sources therefore differs from the number retrieved by the main search.

### 2.3. Study Selection and Interpretation

The search strategy and scope of the review were reviewed and agreed upon by the coauthors. Publications were selected according to their relevance to the review objectives, with emphasis on evidence obtained directly in women with GDM. Preference was given to original studies of women with GDM, particularly those relating to islet autoantibodies, beta-cell function, HLA associations or risk of post-pregnancy diabetes. In addition, reviews and meta-analyses were included to give a broader insight into the available evidence. When studies specifically involving pregnant women were limited, selected research on type 1 diabetes (T1D) and autoimmune diabetes in adults was used to explain possible underlying mechanisms. These findings were clearly distinguished from evidence obtained directly from women with GDM. New molecular biomarkers were considered exploratory when their association with an autoimmune phenotype of GDM had not yet been demonstrated.

The available evidence was summarized and discussed according to the main topics of the review. Table 1 displays whether findings are based on studies in women with GDM, evidence from T1D or adult autoimmune diabetes or preliminary research that needs further investigation.

## 3. Heterogeneity of Gestational Diabetes Mellitus

GDM is a heterogeneous condition in which hyperglycemia may arise from different combinations of insulin resistance and impaired β-cell adaptation. In a small proportion of women, islet autoimmunity adds a further layer of heterogeneity. Importantly, the detection of an islet autoantibody does not necessarily indicate clinically significant autoimmune β-cell disease. Autoantibody status, β-cell functional reserve, and the subsequent clinical course therefore need to be considered separately. Throughout this review, we distinguish between islet-autoantibody positivity, an autoimmune phenotype of GDM, and autoimmune diabetes first recognized during pregnancy. These terms are used as working concepts rather than as formally established diagnostic categories and are summarized in Table 2.

The development of GDM depends on the balance between insulin resistance during pregnancy and the ability of pancreatic β-cells to increase insulin secretion. During normal pregnancy, placental hormones and other factors, including human placental lactogen, placental growth hormone, progesterone, estrogens, prolactin, cortisol, inflammatory cytokines, and growth factors, contribute to changes in maternal insulin sensitivity. Insulin resistance increases particularly during the second and third trimesters. To maintain normal blood glucose levels, pancreatic β-cells adapt by increasing insulin production and secretion. When this response is insufficient to meet the increased metabolic demands of pregnancy, hyperglycemia develops [12,19].

Although women with GDM share the same clinical diagnosis, the underlying mechanisms may differ. Based on these differences, GDM can be described in terms of four main phenotypes: insulin-resistant, insulin-deficient, mixed, and autoimmune [7]. However, these phenotypes are not separate or mutually exclusive conditions. Insulin resistance, β-cell dysfunction, and islet autoimmunity may contribute to different degrees in individual women, and their relative importance may change over time. Importantly, the presence of islet autoantibodies alone does not necessarily indicate impaired β-cell function. Therefore, isolated autoantibody positivity should be distinguished from cases in which autoimmunity is accompanied by reduced β-cell function.

The most common form of GDM is the insulin-resistant phenotype. It is most often associated with maternal obesity, excessive gestational weight gain, pre-pregnancy dyslipidemia and chronic low-grade inflammation. Women with this phenotype typically have relatively intact β-cell function, but their β-cells are unable to compensate fully for pregnancy-induced insulin resistance. Higher circulating levels of proinflammatory cytokines, adipokines and free fatty acids further aggravate insulin resistance and maternal hyperglycemia [7].

The insulin-deficient phenotype is characterized mainly by pancreatic β-cell dysfunction in the setting of moderate insulin resistance. These women tend to be younger, have a lower pre-pregnancy body mass index (BMI) and show decreased endogenous insulin secretion during the oral glucose tolerance test (OGTT). In this subgroup, the dominant factors are not insulin resistance but genetic and other determinants affecting β-cell development and secretory capacity [7,12].

A third subgroup consists of women with a mixed phenotype in which both insulin resistance and impaired insulin secretion contribute to gestational hyperglycemia. This phenotype illustrates the complex interaction between genetic predisposition and metabolic stress during pregnancy and represents a substantial proportion of women diagnosed with GDM.

The autoimmune phenotype is the least common but biologically one of the most distinctive forms of GDM. Unlike the other phenotypes, it is associated with immune-mediated dysfunction of pancreatic β-cells, reflected by circulating autoantibodies against islet antigens. Women with this phenotype often have relatively preserved insulin sensitivity for the degree of hyperglycemia, a lower pre-pregnancy BMI, an earlier need for insulin therapy and a higher frequency of other autoimmune diseases, such as autoimmune thyroid disease and celiac disease [13,20].

Importantly, islet autoimmunity may occur alongside different metabolic features of GDM. For example, a woman with obesity and marked insulin resistance may be positive for GADA, while a lean woman with reduced insulin secretion may have no detectable islet autoantibodies. Therefore, the differences presented in Table 3 should be viewed as general tendencies rather than strict diagnostic criteria. Recognizing these differences is clinically important because glucose levels alone do not fully reflect the biological diversity of GDM. Considering metabolic, immunological, and genetic characteristics alongside glucose measurements may help improve the classification of GDM and support more individualized patient care [8].

An important question is whether the autoimmune phenotype represents an independent nosological entity or the first clinical manifestation of pre-existing autoimmune diabetes. In several respects, the autoimmune phenotype resembles latent autoimmune diabetes in adults (LADA): islet autoantibodies, a gradual decline in β-cell reserve and a progressive need for insulin are observed. However, LADA is defined outside pregnancy, and its diagnostic criteria cannot be applied directly to GDM [21]. From a clinical perspective, the autoimmune phenotype covers a spectrum. In some women, progression to classical T1D occurs after delivery; in others, the course is slower and resembles LADA; and in still others, antibodies disappear postpartum without apparent metabolic consequences. For this reason, some authors regard the more advanced forms as pregnancy-unmasked autoimmune diabetes rather than as a separate form of diabetes [16,21,22]. The available data therefore support the existence of an autoimmune subgroup but do not yet resolve its nosological position; the continuum model appears to reflect current evidence best.

## 4. Immunopathophysiology of the Autoimmune Phenotype of GDM

### 4.1. Pregnancy as a Metabolic Stress Test

Pregnancy is a state of metabolic adaptation that ensures a continuous supply of nutrients to the developing fetus while maintaining maternal glucose homeostasis. Physiological insulin resistance develops because of placental hormone production and favors the preferential transfer of glucose to the fetus. As insulin resistance increases during the second and third trimesters, maternal β-cells must increase insulin secretion to maintain glucose homeostasis. This increased secretory demand places a physiological burden on β-cell functional reserve and may reveal an inability to compensate adequately for pregnancy-induced insulin resistance [12,19].

**Figure 1:**
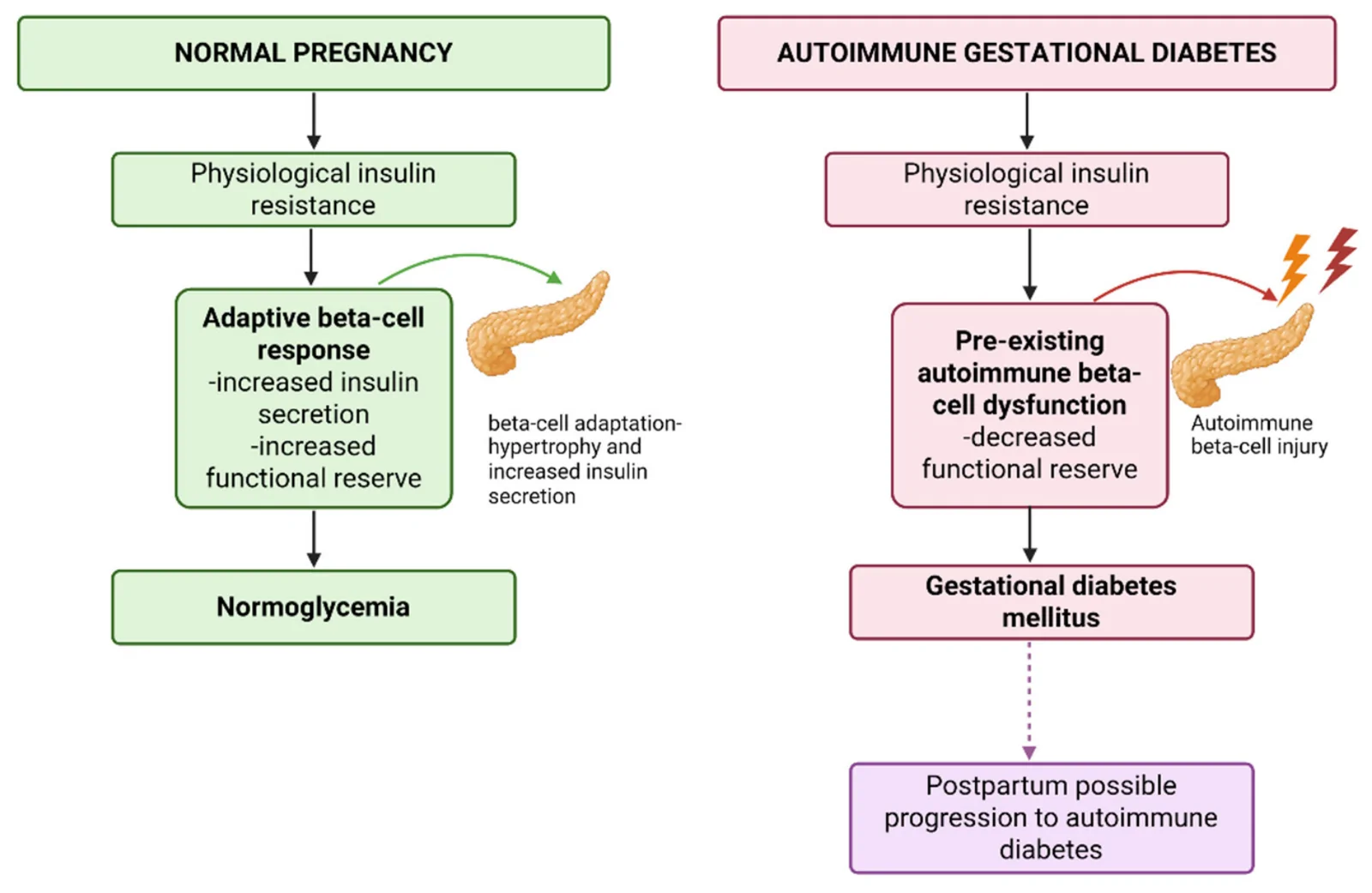
Proposed model of β-cell adaptation and compensation failure during pregnancy. In normal pregnancy, physiological insulin resistance is accompanied by adaptive increases in β-cell function and insulin secretion, thereby maintaining normoglycemia. In women with an autoimmune phenotype of gestational diabetes mellitus (GDM), reduced functional β-cell reserve associated with islet autoimmunity may limit the compensatory response to pregnancy-induced insulin resistance, resulting in gestational hyperglycemia. After delivery, a subset of women may show persistent or progressive autoimmune β-cell dysfunction and subsequently develop autoimmune diabetes. The model should be interpreted across the preconception/early-pregnancy, mid-to-late-pregnancy and postpartum periods. These are conceptual stages rather than an established longitudinal trajectory. The timing of islet-autoantibody appearance relative to changes in C-peptide remains uncertain; the diagram therefore does not imply a defined onset or intensity of immune attack, or a uniform turning point in β-cell functional decline. *Created in BioRender. Yanachkova, V. (2026) BioRender.com/0carbhm*.

In normal pregnancy, pancreatic β-cells respond with adaptive mechanisms that include increased stimulated insulin secretion, increased insulin biosynthesis and functional hypertrophy. This process is accompanied by expansion of functional β-cell mass, which preserves maternal normoglycemia despite peripheral insulin resistance [19,23]. The adaptive capacity of β-cells varies between women and influences their ability to maintain normal glucose levels during pregnancy. In women with an autoimmune phenotype, limited β-cell reserve may impair compensation for pregnancy-induced insulin resistance. Pregnancy may therefore act as a physiological metabolic stress test, making previously compensated dysfunction clinically apparent [9,10,16] (Figure 1). This interpretation does not establish when islet autoimmunity began or whether pregnancy changes its subsequent course. The stress-test framework describes the relationship between increased metabolic demand and β-cell functional reserve. It should not be interpreted as implying that pregnancy is immunologically neutral. Metabolic unmasking of a pre-existing deficit and pregnancy-related modification of the autoimmune process are compatible possibilities. Pregnancy-associated immune adaptation could potentially modify pre-existing islet autoimmunity, while changes after delivery could alter its subsequent trajectory. These possibilities remain hypotheses in islet-autoantibody-positive GDM; observations in other autoimmune diseases cannot establish that the same processes occur in this population. The lower pooled frequency of autoantibody positivity reported in postpartum samples [17] requires cautious interpretation. Comparisons between antenatal and postpartum prevalence do not necessarily represent changes within the same women. Even when antibody disappearance is documented longitudinally, it does not by itself demonstrate recovery of β-cell function. Thus, antibody trajectories should be interpreted according to insulin secretion, insulin sensitivity and glycemic outcomes. Evidence supporting a revelatory effect would be the finding of autoantibodies and poor β-cell reserve before conception, followed by hyperglycemia as gestational insulin resistance increases. Evidence of modifying effect would require changes in islet-directed immunity or β-cell functional trajectories above and beyond what is expected from metabolic demand. Studies beginning before conception, with serial standardized autoantibody measurements, assessment of stimulated C-peptide relative to insulin sensitivity, and follow-up through pregnancy and postpartum would help distinguish these patterns. Comparable nonpregnant women would strengthen interpretation, although observational findings alone would not establish causality. Pregnancy as a metabolic stress test is therefore presented as an interpretative framework whose immunological boundaries remain to be defined.

### 4.2. Cellular Immune Mechanisms

Much of the detailed cellular mechanism described below has been established in T1D and is extrapolated to pregnancy; direct evidence in women with GDM is limited (Table 1). In T1D, cellular immune mechanisms are the dominant mediators of β-cell destruction. Autoreactive CD4^+^ helper T lymphocytes recognize β-cell antigens presented by antigen-presenting cells via major histocompatibility complex class II molecules and orchestrate adaptive immune responses by stimulating cytokine production and activating cytotoxic lymphocytes. Cytotoxic CD8^+^ T cells infiltrate the pancreatic islets and directly induce β-cell apoptosis [24,25]. Although T lymphocytes are the principal effector cells, B lymphocytes also contribute to pathogenesis. B cells not only produce islet autoantibodies but also act as antigen-presenting cells that support autoreactive T-cell responses. The interaction between T and B lymphocytes sustains chronic islet inflammation and the progressive loss of functional β-cell mass [11].

In experimental models and studies of T1D, proinflammatory cytokines contribute to β-cell dysfunction and injury. IL-1β, IFN-γ and TNF-α can impair insulin secretion, alter insulin gene expression and activate pathways involved in cellular stress and apoptosis [26,27]. Prolonged exposure to inflammatory mediators may also promote endoplasmic reticulum stress, oxidative stress and mitochondrial dysfunction. These mechanisms may be relevant to islet-autoantibody-positive GDM, but their contribution has not been established directly [26,27].

Also implicated has been immune dysregulation. Proinflammatory T-helper 17 (Th17) lymphocytes and regulatory T cells (Treg) are unbalanced in T1D as reported in several studies. Reduced Treg activity decreases peripheral immune tolerance, while enhanced Th17 responses promote the production of IL-17 and other inflammatory mediators, leading to chronic immune activation against β-cells [25,28]. The autoimmune response may broaden over time to involve additional islet antigens. In the TEDDY study, children with increased genetic susceptibility to T1D were followed for the sequential appearance of distinct islet autoantibodies and progression to clinical diabetes [29]. This pattern is consistent with spreading of the autoimmune response, but does not directly demonstrate the cellular mechanism of epitope spreading. Its relevance to women with islet-autoantibody-positive GDM remains an extrapolation requiring pregnancy-specific investigation. Pregnancy itself requires profound immunological adaptation to maintain maternal–fetal tolerance, and it is plausible that it modifies pre-existing islet autoimmunity. However, pregnancy-specific evidence linking Th17/Treg imbalance or cytokine profiles directly to antibody-positive GDM remains sparse. Moreover, pregnancy and obesity are themselves associated with low-grade inflammation, so systemic inflammatory markers in unselected GDM cohorts cannot be taken as evidence of islet-specific autoimmunity.

**Figure 2:**
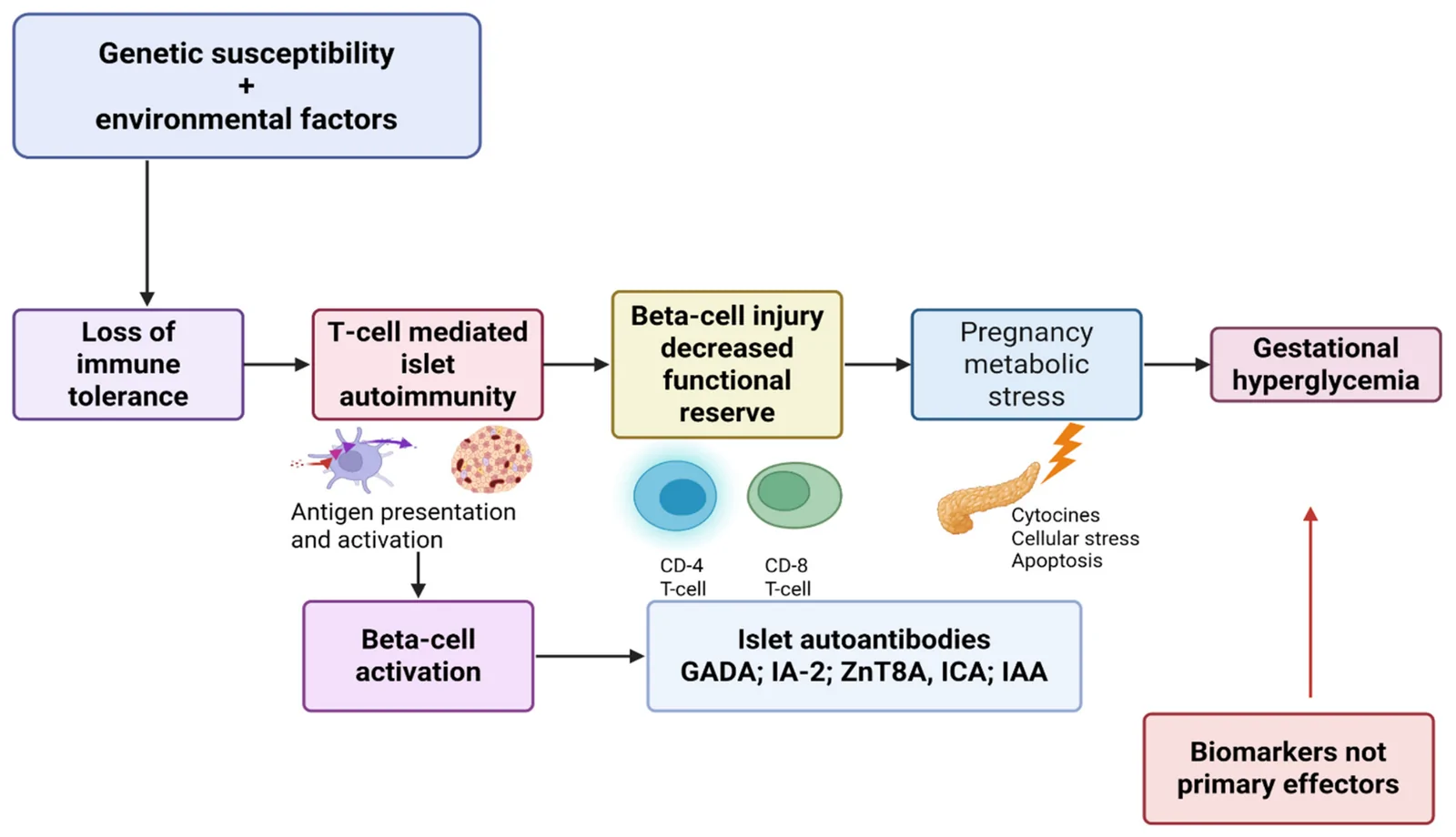
Proposed immune mechanisms underlying the autoimmune phenotype of gestational diabetes mellitus. Genetic predisposition and environmental factors causing β-cell damage and reduced functional β-cell reserve may lead to loss of immune tolerance and activation of islet-directed cellular immunity. Pregnancy-related metabolic stress may unmask insufficient β-cell compensation and contribute to gestational hyperglycemia. B-cell activation and the appearance of islet autoantibodies, including GADA, IA-2A, ZnT8A, ICA and IAA, serve primarily as biomarkers of islet autoimmunity rather than direct mediators of β-cell destruction. The proposed model is derived largely from mechanisms established in type 1 diabetes, as pregnancy-specific mechanistic evidence in GDM remains limited. *Created in BioRender. Yanachkova, V. (2026) BioRender.com/jy3vpcn*.

Circulating islet autoantibodies are primarily markers of islet-directed autoimmunity rather than direct mediators of β-cell destruction [11,24,29]. In T1D, β-cell injury is driven mainly by cellular immune mechanisms. In women with GDM, antibody positivity alone does not establish ongoing β-cell destruction, the rate of functional decline or a validated disease stage. Antibody findings should therefore be interpreted together with their persistence, the number of antibodies detected, β-cell function and clinical course (Figure 2).

### 4.3. Genetic and Environmental Determinants

Genetic susceptibility and environmental exposures are involved in the development of T1D and may also be relevant to islet autoimmunity detected during pregnancy. However, their specific contribution to the autoimmune phenotype of GDM remains incompletely defined [12,24]. Genetic susceptibility is the best-established risk factor for autoimmune diabetes and is attributed mainly to polymorphisms of the human leukocyte antigen (HLA) region on chromosome 6p21, particularly class II alleles involved in antigen presentation to CD4^+^ T lymphocytes. The HLA-DR3-DQ2 and HLA-DR4-DQ8 haplotypes are most strongly associated with T1D, and HLA-DQB1 genotypes have been associated with islet autoantibodies and postpartum diabetes in women with GDM [24,30]. These HLA molecules influence the efficiency with which β-cell peptides are presented to autoreactive T cells; some variants bind β-cell peptides with greater affinity, favoring adaptive immune responses and loss of tolerance, whereas protective alleles appear to promote immune regulation. Routine HLA genotyping is not currently recommended in clinical practice, but knowledge of genetic predisposition provides insight into the biological mechanisms underlying the autoimmune phenotype [24,30].

Several non-HLA genes involved in immune regulation, β-cell function and inflammatory signaling have also been linked to autoimmune diabetes, including PTPN22, CTLA4, IL2RA, IFIH1 and SH2B3. They are believed to modulate immune tolerance and T-cell activation [24]. Although the effect of individual non-HLA loci is small relative to HLA class II genes, their additive effects may be important. Future polygenic risk assessment may improve identification of women at risk before pregnancy, although pregnancy-specific data are currently lacking.

Genetic susceptibility alone does not explain the development of autoimmune diabetes. Several environmental factors have been proposed as triggers. Viral infections, particularly enteroviruses such as Coxsackievirus B, have long been implicated in autoimmune β-cell damage through molecular mimicry, bystander activation and persistent infection of the islets. Experimental studies have shown that viral infection can promote β-cell antigen presentation and local inflammatory responses that facilitate activation of autoreactive T lymphocytes [24].

The gut microbiome has also been considered among the environmental factors potentially involved in T1D pathogenesis, with proposed effects on immune regulation [24]. Its relevance to islet-autoantibody-positive GDM remains uncertain. Microbiome alterations observed in GDM as a whole should not be interpreted as evidence of islet-directed autoimmunity, because the metabolic phenotype and the presence of autoimmunity need to be distinguished. Within the framework of this review, the microbiome is therefore considered a candidate modifier requiring investigation in women characterized by both islet-autoantibody status and β-cell function, rather than an established mechanism of autoimmune GDM.

Other environmental factors, such as vitamin D deficiency, smoking, dietary exposures and pollutants, have also been explored. Vitamin D has immunomodulatory effects on dendritic-cell maturation, T-cell differentiation and regulatory T-cell activity, but a direct causal relationship has not been demonstrated [26,28]. Genetic and environmental factors do not act independently but interact continuously throughout life to shape immune function (Table 4).

## 5. Biomarkers of the Autoimmune Phenotype of GDM

Identification of the autoimmune phenotype relies, in addition to clinical features and the degree of hyperglycemia, on biomarkers reflecting immune-mediated β-cell dysfunction. Among those currently available, diabetes-associated islet autoantibodies remain the most extensively studied; in T1D they appear months or even years before the onset of overt disease [11,24]. Beyond autoantibodies, measurement of fasting and stimulated C-peptide provides information on residual β-cell function and endogenous insulin secretion, and metabolic indices derived from the OGTT can further characterize insulin secretory capacity and insulin sensitivity. Immunological and metabolic biomarkers therefore provide a more comprehensive assessment of the phenotype than either approach alone. No single laboratory test is sufficiently sensitive or specific to identify all women with the autoimmune phenotype, and current evidence supports a multimarker strategy that combines autoantibodies with clinical features and measures of β-cell function [11,12,31]. The main biomarkers are summarized in Table 5.

### 5.1. How Common Is Islet Autoimmunity in GDM?

Reported prevalence has varied widely because studies differ in GDM diagnostic criteria, ethnicity, gestational age at sampling, autoantibody panels, laboratory assays, positivity thresholds and duration of follow-up. Cohort-specific estimates point to the significance of the population and laboratory context. In the Lund cohort, Nilsson et al. detected GADA in 23 of 385 women with GDM (approximately 6%), using a radioimmunoprecipitation assay with radiolabelled human GAD65 [10]. In the Finnish cohort reported by Luiro et al., GADA were detected in first-trimester samples from 21 of 350 women with available GADA measurements (6.0%), using a specific radiobinding assay [14]. These estimates describe the respective cohorts and should not be interpreted as a general prevalence range applicable across populations, sampling periods and assay platforms.

A recent systematic review and meta-analysis of 21 studies estimated a pooled prevalence of any islet autoantibody of approximately 11%, with extreme between-study heterogeneity, a markedly higher frequency than in pregnant women without GDM, and lower pooled positivity in postpartum samples [17]. These findings confirm the enrichment of islet autoimmunity in GDM while showing that prevalence estimates depend strongly on assay and context. The lower postpartum prevalence also indicates that an antibody-positive result obtained during pregnancy should not automatically be equated with progressive autoimmune diabetes.

### 5.2. Glutamic Acid Decarboxylase Autoantibodies (GADA)

Autoantibodies against glutamic acid decarboxylase (GADA) are the most common and clinically informative serological markers of autoimmune diabetes in adults. They are directed against the 65-kDa isoform of glutamic acid decarboxylase (GAD65) and reflect loss of immune tolerance to one of the major intracellular antigens of pancreatic β-cells [11]. Within the spectrum of GDM, GADA is the predominant biomarker of the autoimmune phenotype, although its frequency varies with ethnicity, laboratory methodology and diagnostic criteria [9,14]. GADA measurements are not affected by exogenous insulin therapy and show good analytical reproducibility with standardized immunoassays, which makes GADA the laboratory marker of choice when the autoimmune phenotype is suspected.

A positive GADA test should not, however, be interpreted as evidence of ongoing β-cell destruction per se. It indicates an adaptive immune response directed against β-cell antigens. The probability of progressive β-cell dysfunction increases when GADA is persistent, clearly positive, accompanied by other diabetes-related autoantibodies or associated with reduced endogenous insulin secretion [9,11,14]. Among currently available islet autoantibodies, GADA is the most practical first-line marker when autoimmune diabetes is suspected in a woman with GDM. However, GADA positivity alone does not establish clinically significant autoimmune β-cell dysfunction.

### 5.3. Complementary Islet Autoantibodies

In addition to GADA, other diabetes-associated autoantibodies (IA-2A, ZnT8A, ICA and IAA) provide information about the extent of β-cell autoimmunity. These antibodies differ in their biological targets, prevalence and clinical significance. Only a proportion of women with the autoimmune phenotype are detected by any single marker, and combined antibody profiling considerably improves characterization [14,15]. In autoimmune diabetes, the sequential appearance of antibodies against different islet antigens is consistent with spreading of the autoimmune response [29]. Multiple autoantibodies have also been associated with a higher risk of subsequent T1D in GDM cohorts [14,15], although the underlying immune process has not been characterized to the same extent in women with GDM.

**IA-2A**—IA-2A are directed against insulinoma-associated antigen-2 (IA-2), a transmembrane protein localized in the dense-core secretory granules of β-cells. Although less common than GADA, IA-2A is considered a highly specific marker of autoimmune diabetes and is associated with more advanced β-cell autoimmunity [11]. IA-2A appear less frequently than GADA in women with GDM, although reported frequencies vary between populations and studies [9,14]. Women positive for both GADA and IA-2A tend to show greater impairment of insulin secretion than those positive for GADA alone, suggesting that IA-2A identifies a subgroup with more extensive autoimmune β-cell damage [9,14].

**ZnT8A**—ZnT8A recognize zinc transporter 8, a β-cell-specific membrane protein that transports zinc ions into insulin secretory granules. Zinc is required for insulin crystallization and storage, and ZnT8 is therefore an important component of normal β-cell physiology [32,33]. ZnT8A appear to be relatively uncommon in GDM, and pregnancy-specific data remain limited [32]. Their clinical value lies in improving the sensitivity of autoimmune assessment when combined with GADA and IA-2A, and they may detect autoimmunity in women negative for other antibodies [32]. The incremental predictive value of ZnT8A in pregnancy has not yet been established prospectively.

**ICA**—ICA were the first serological markers of autoimmune diabetes and were central to the development of the concept of islet autoimmunity [34]. ICA represent a heterogeneous group of antibodies against several cytoplasmic islet-cell antigens and are detected by indirect immunofluorescence. Antigen-specific assays have proved more sensitive, specific and reproducible, and the diagnostic role of ICA in modern practice has therefore declined; today, ICA measurements are largely limited to research and long-term epidemiological studies [11]. Because ICA results depend heavily on laboratory expertise, older ICA-based prevalence estimates are difficult to compare with contemporary biochemical assays and probably contribute to the heterogeneity observed between studies [17].

**IAA**—IAA are directed against endogenous insulin and are among the earliest markers of autoimmune diabetes in childhood. Their interpretation in adults is considerably more difficult because insulin therapy itself induces antibody formation. IAA measurements are therefore of limited value in women who have already started insulin during pregnancy. Isolated IAA positivity is rare in GDM and contributes little to routine phenotyping; however, when detected before insulin therapy or together with other islet autoantibodies, IAA may provide supportive information [11].

### 5.4. Number, Persistence and Titer of Autoantibodies

Islet autoantibodies identify islet-directed autoimmunity, but their presence alone does not establish that autoimmunity is responsible for gestational hyperglycemia or that clinically significant β-cell dysfunction is present. Their interpretation therefore requires consideration of antibody number, persistence and titer together with β-cell function and the clinical course. In some women, the antibodies were probably present before pregnancy; because prospective studies starting before conception are lacking, the timing of seroconversion remains unknown. Long-term follow-up of women with GDM has shown that ICA and GADA detected during pregnancy predict subsequent T1D, with the highest risk in the presence of multiple autoantibodies. In a prospective multicenter German cohort, the risk of T1D after GDM increased steeply with the number of antibodies detected [15], and in a Finnish cohort followed for 23 years, antibody profiles measured during pregnancy identified women who developed T1D many years later [14].

Diagnostic and prognostic interpretation should therefore take into account not only the presence but also the number, combination, titer and persistence of autoantibodies. A single positive autoantibody, especially at low titer, should be confirmed by repeat testing, as it may represent a transient or nonspecific finding; in a Belgian cohort, only a minority of antibody-positive women remained positive at long-term follow-up [35]. Confirmation should ideally be performed in a laboratory participating in the Islet Autoantibody Standardization Program [36]. Persistence of two or more islet autoantibodies is a significantly stronger marker of progressive autoimmune β-cell failure. In children with confirmed multiple autoantibodies, prospective cohorts indicate a very high long-term risk of clinical T1D [22]. This classification should not, however, be extrapolated automatically to pregnant women, as validated risk thresholds for the autoimmune phenotype of GDM do not exist. The number of antibodies in pregnancy should therefore be regarded as a prognostic marker rather than as an independent diagnostic criterion or basis for defining a separate disease subtype.

### 5.5. The Role of C-Peptide

C-peptide testing complements autoantibody assessment by providing a measure of endogenous insulin secretion. It can help assess β-cell function, but it does not directly measure β-cell mass or establish an autoimmune cause of impaired secretion. In women with previous GDM, GADA positivity has been associated with lower fasting C-peptide and a lower disposition index than in antibody-negative women [20].

During pregnancy, fasting C-peptide should be interpreted cautiously, because physiological insulin resistance leads to compensatory increases in endogenous insulin secretion, and fasting C-peptide may remain preserved in early autoimmune diabetes despite ongoing immune-mediated injury. Stimulated C-peptide, measured during an OGTT or mixed-meal tolerance test, therefore reflects β-cell reserve more accurately. Lower stimulated C-peptide concentrations indicate reduced secretory capacity and may help identify women with the autoimmune phenotype before complete β-cell failure [20]. The coexistence of GADA positivity with low or progressively declining C-peptide may indicate more advanced β-cell dysfunction and may identify women who warrant closer postpartum surveillance [20]. Dynamic indices such as the insulinogenic index and disposition index relate secretion to the glucose stimulus and to insulin sensitivity; they are valuable research tools but have not been standardized for routine pregnancy care.

## 6. Biomarkers and New Approaches

Because broadening immune reactivity toward different β-cell antigens accompanies the decline in insulin secretory capacity, autoantibody combinations characterize autoimmune β-cell dysfunction better than any single marker [14,15]. Autoantibodies alone, however, provide limited information on disease activity and residual β-cell function [11,31], and future classification is likely to combine antibody profiles with measures of β-cell function, genetic susceptibility, clinical features and, potentially, molecular biomarkers.

Rapid advances in genomic technologies have improved understanding of the hereditary predisposition to autoimmune diabetes. Although HLA genotyping remains primarily a research tool, combining HLA class II genotypes with non-HLA susceptibility variants in polygenic risk scores may eventually help identify women at increased risk before pregnancy, provided such scores are validated across diverse ancestries and in pregnancy cohorts.

Characterization of immune-cell populations may also add information beyond standard autoantibody testing. In autoimmune diabetes, alterations in regulatory T cells, activated CD8^+^ cytotoxic T lymphocytes, follicular helper T cells and B-cell subsets have been associated with β-cell autoimmunity, and circulating cytokine profiles, including IL-17, IL-6, TNF-α, IFN-γ and IL-10, may relate more closely to disease activity than autoantibody titers alone [25,28]. These observations, however, derive mainly from T1D.

Several novel molecular biomarkers are also under investigation. Circulating microRNAs are of particular interest because of their involvement in the regulation of immune activation, inflammation and β-cell survival; several microRNA signatures have been associated with GDM, but their specific role in the autoimmune phenotype has not been studied [37]. Metabolomic profiling offers another approach, as changes in amino-acid and lipid metabolism and mitochondrial function may precede clinically meaningful hyperglycemia [38]. Proteomic analyses and extracellular-vesicle profiling are further potential sources of biomarkers reflecting β-cell stress and immune activation [39].

The principal limitation of all these approaches is specificity. Most studies have examined GDM as a whole rather than women defined by standardized autoantibody profiles and β-cell function. None of these biomarkers have yet been incorporated into clinical practice, and they should currently be regarded as discovery platforms. Their future role will depend on whether they improve prediction beyond antibody specificity, persistence, C-peptide and conventional clinical variables (Table 6).

## 7. Clinical Perspective

Because conventional criteria classify GDM solely by glycemic thresholds, women with different pathophysiological processes are usually managed with identical strategies. Although the autoimmune phenotype accounts for a relatively small proportion of GDM, its recognition may influence clinical decisions in several ways. Women with autoimmune β-cell dysfunction often present with features that differ from classical insulin-resistant GDM and that may help clinicians identify patients who need further evaluation. They tend to be younger and leaner, with less insulin resistance and a greater requirement for insulin treatment. A personal or family history of autoimmunity, including autoimmune thyroid disease, celiac disease, vitiligo or T1D, may further increase clinical suspicion [9,20]. None of these features, however, are specific enough to establish the diagnosis: obesity does not exclude autoimmune diabetes, and insulin treatment may reflect local treatment thresholds or the severity of hyperglycemia rather than autoimmune β-cell failure. These features should be regarded as indicators that support further evaluation when considered together with laboratory findings.

### 7.1. Who Should Be Tested?

Routine islet-autoantibody testing is not currently part of standard GDM assessment in major international guidelines [1,3,4,5,6]. Evidence is currently insufficient to support universal islet-autoantibody screening in women with GDM, particularly because the clinical significance of isolated or transient autoantibody positivity remains uncertain and pregnancy-specific evidence that screening improves clinical outcomes is lacking. Selective testing may nevertheless be considered in women with clinical features suggesting impaired β-cell reserve rather than predominantly insulin resistance (Table 7). GADA is the most practical first-line islet autoantibody in this setting, with IA-2A and/or ZnT8A providing additional information when clinical suspicion remains high. This approach is based largely on evidence and practice from adult autoimmune diabetes [21,31,40] and has not been prospectively validated in women with GDM.

### 7.2. Interpretation of Findings

**Figure 3:**
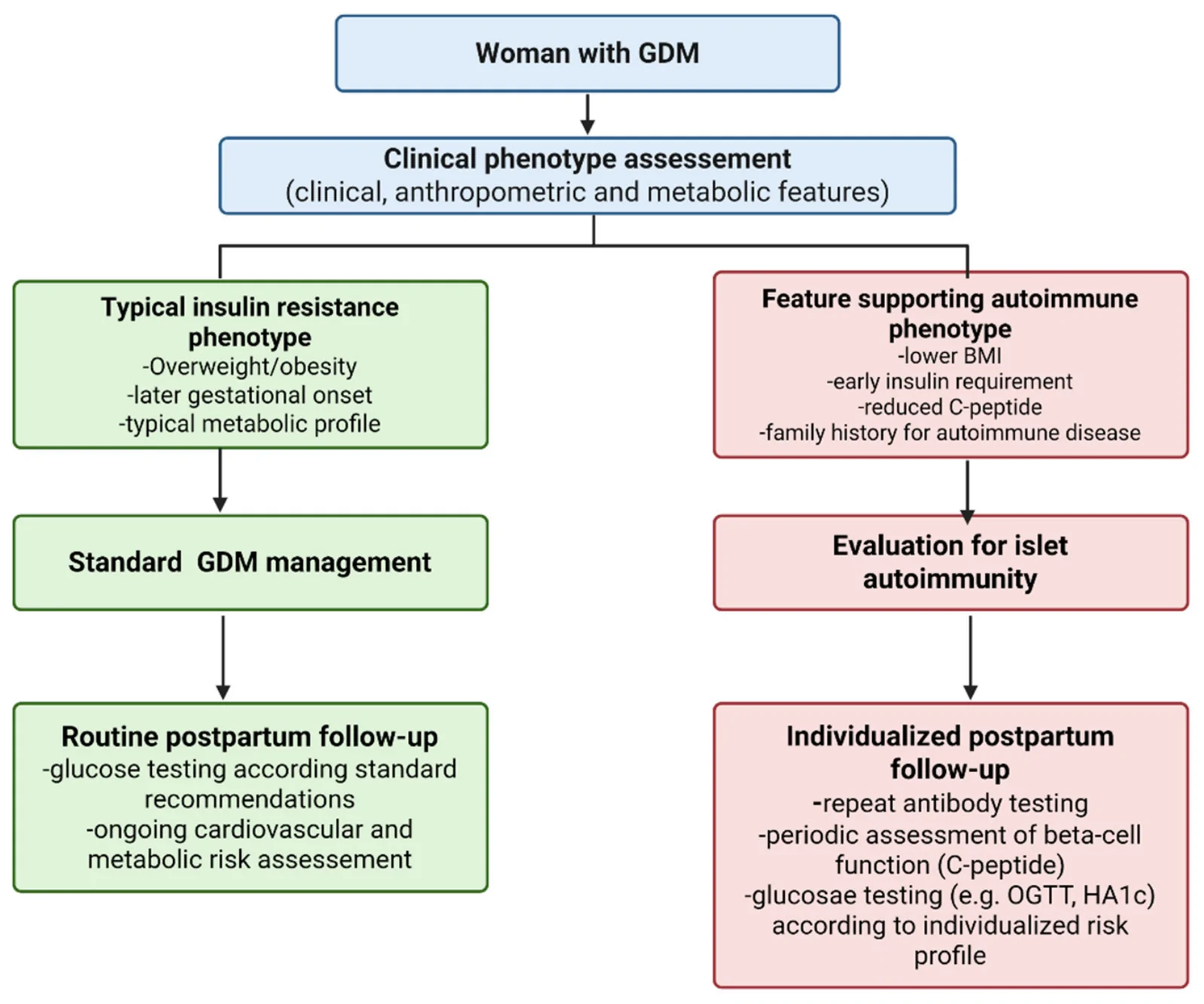
Proposed phenotype-oriented pathway for the evaluation of islet autoimmunity in women with gestational diabetes mellitus. Women with GDM may be considered for further evaluation according to their clinical, anthropometric and metabolic phenotype. Features suggesting impaired β-cell reserve or possible autoimmune involvement may prompt selective assessment of GADA, with additional IA-2A and/or ZnT8A testing when clinically appropriate, together with evaluation of endogenous insulin secretion using C-peptide. The combination of clinical characteristics, autoantibody profile and β-cell function may help identify women who warrant individualized postpartum metabolic surveillance. This pathway represents a conceptual, evidence-informed approach for clinical evaluation and future research and has not been prospectively validated as a screening or management algorithm in women with GDM. *Created in BioRender. Yanachkova, V. (2026) BioRender.com/cxxnt1v*.

The proposed approach starts with the diagnosis of GDM according to current recommendations, followed by assessment of clinical features that may suggest insulin deficiency. In selected women, further evaluation may include GADA, C-peptide and, where appropriate, additional islet autoantibodies (Figure 3). The approach combines observational findings in GDM with diagnostic practices used in adult autoimmune diabetes. It has not been prospectively validated in GDM, and its effects on diagnostic accuracy, clinical outcomes and cost-effectiveness remain unknown. It is therefore presented as a framework for individualized specialist assessment and future research.

Results should be interpreted along a gradient of certainty rather than as a simple positive/negative dichotomy (Table 8). The same GADA result has different implications in a woman with declining C-peptide, early insulin dependence and postpartum dysglycemia than in a woman with preserved secretion and normal postpartum glucose tolerance. Conversely, an insulin-deficient phenotype without detectable antibodies may reflect non-autoimmune β-cell dysfunction, monogenic diabetes, antibody-negative autoimmune diabetes or limitations of the antibody panel.

### 7.3. Management During Pregnancy

There is currently no evidence-based immune-directed treatment for the autoimmune phenotype of GDM, and no antibody-specific glycemic targets have been validated. Management should therefore follow established diabetes-in-pregnancy principles, including medical nutrition therapy, glucose monitoring and pharmacological treatment, usually insulin, when glycemic targets are not achieved [1,4,5,6]. Insulin may be required earlier in women with limited β-cell reserve, but insulin requirement reflects insufficient endogenous secretion and/or insulin resistance and is not in itself proof of autoimmunity. The immediate value of recognizing convincing autoimmunity lies in more accurate classification, anticipation of insulin deficiency and a lower risk that evolving T1D, particularly with ketosis or marked hyperglycemia, is managed as routine GDM [41].

### 7.4. Associated Thyroid Autoimmunity

Associated thyroid autoimmunity is relevant to the assessment of women with GDM, but evidence concerning its relationship with islet autoimmunity requires careful interpretation. In a study evaluating thyroid and GAD65 antibodies, thyroid autoimmunity did not differ significantly between women with GDM and controls during pregnancy, whereas a higher prevalence was observed in a separate group of women with previous GDM. However, only one participant had concurrent GAD65- and TPO-antibody positivity, limiting conclusions about clustering of thyroid and islet autoimmunity [42].

These findings distinguish two questions: whether thyroid autoimmunity is associated with GDM overall, and whether it specifically accompanies autoimmune β-cell dysfunction. Evidence addressing the former should not be taken as proof of the latter. Similarly, shared HLA susceptibility is a possible explanation for autoimmune clustering, but its contribution to the coexistence of thyroid and islet autoimmunity in GDM remains unconfirmed. Clinically, a history of autoimmune thyroid disease may contribute to the broader assessment when autoimmune diabetes is suspected, but should be interpreted together with islet-autoantibody profiles, β-cell function and the clinical course. Thyroid-function testing and thyroid-autoantibody assessment should follow current guidance for pregnancy and the postpartum period [43].

### 7.5. Postpartum Follow-Up

Postpartum follow-up is essential for all women with GDM, and particularly for those with the autoimmune phenotype because of their risk of progression to autoimmune diabetes. Unlike classical GDM, in which the main long-term risk is type 2 diabetes, the autoimmune phenotype may be followed by gradual or rapid loss of β-cell function, which can emerge years after the index pregnancy [9,14]. A 75-g OGTT is recommended in the early postpartum period (commonly 4–12 weeks after delivery), complemented where appropriate by HbA1c, C-peptide and repeat islet-autoantibody testing [1,5,6]. Because antibody status can change after delivery [17,35], a single gestational measurement should not be treated as a permanent phenotype. In women with persistent GADA, especially in the presence of more than one autoantibody or declining C-peptide, periodic (for example, annual) monitoring of glycemic status and β-cell function appears advisable, although intervals have not been validated specifically after GDM. Consensus guidance for monitoring adults with confirmed islet autoantibodies provides a useful reference [44]. Such follow-up may help identify progressive insulin deficiency, but the clinical benefit of additional antibody- and C-peptide-based surveillance has not been demonstrated specifically after GDM. The intensity of follow-up should be individualized, taking into account antibody persistence, β-cell function and glycemic status. Risk communication should distinguish relative risk from an individual woman’s absolute risk.

### 7.6. Implications for the Offspring

Particular care is required in the interpretation of autoantibodies detected in a newborn. GADA, IA-2A, ICA and insulin antibodies of maternal origin can cross the placenta and be detected in cord blood or during the first months of life; they usually disappear within the first 6–9 months. A positive result immediately after birth therefore does not demonstrate an autoimmune process in the child, whereas persistent or newly appearing autoantibodies after clearance of maternal immunoglobulins are more likely to reflect active autoimmunity. Testing for islet autoantibodies in cord blood or immediately after birth is not a suitable method for assessing long-term T1D risk [22]. Risk estimates derived from families affected by established T1D should not be directly applied to children of women with islet-autoantibody-positive GDM. Maternal autoantibody positivity alone does not establish an equivalent maternal disease phenotype or a corresponding risk in the offspring. Reliable absolute risk estimates specific to this population remain unavailable, and counselling should explicitly acknowledge this uncertainty.

Routine testing of all children born to mothers with autoimmune GDM cannot currently be recommended on the basis of the maternal diagnosis alone. Screening may be considered when the mother subsequently develops confirmed T1D, when other first-degree relatives have T1D, in the presence of multiple familial autoimmunities, or within structured screening programs; any positive result should be confirmed in a standardized laboratory and followed by specialized surveillance [22,40].

## 8. Limitations and Future Directions

### 8.1. Limitations of the Available Evidence

The evidence concerning islet autoimmunity in GDM has several limitations. Persistent islet autoimmunity is relatively uncommon, and even large studies may include only small antibody-positive subgroups. Consequently, estimates of clinical differences and subsequent diabetes risk may be imprecise. Study populations also differ in ethnicity, maternal age, BMI, GDM diagnostic criteria, timing of diagnosis, autoantibody panels, and follow-up duration. These differences limit comparability and are responsible for the heterogeneity found in pooled analyzes [17]. Another problem is variation among laboratories. Assay platforms, positivity thresholds, and procedures for confirming low-positive results are not uniform. Although international standardization has improved autoantibody measurement, results are not fully interchangeable across studies [11,36]. A single low-positive result, persistent positivity, and multiple autoantibodies should therefore not be treated as equivalent findings. Most available studies are observational and establish associations rather than causal relationships. Follow-up periods vary, and relatively few studies provide detailed metabolic and immunological measurements extending beyond 10 years. Moreover, limited sampling before conception makes it difficult to establish whether autoimmunity precedes pregnancy, becomes detectable during gestation, or changes after delivery. These limitations constrain interpretation of pregnancy as a revelatory or modifying influence.

A further difficulty is the absence of a widely accepted definition of autoimmune GDM. Isolated seropositivity, autoimmunity accompanied by impaired β-cell function, and autoimmune diabetes first recognized during pregnancy may be grouped together despite their different implications. Much of the proposed cellular pathophysiology is extrapolated from T1D rather than demonstrated directly in women with GDM. Emerging genetic, immune, and molecular biomarkers remain exploratory and have not been validated for this specific phenotype [37,38,39].

### 8.2. Limitations of the Present Review

The present review also has methodological limitations. Database searching was restricted to PubMed/MEDLINE; Embase and Scopus were not searched. Relevant studies indexed in other databases may therefore have been missed. Reference-list screening and targeted searches supplemented the main search but did not ensure comprehensive coverage.

Publication selection and interpretation were narrative and may have introduced selection bias. The review does not provide a formal study-level assessment of methodological quality or risk of bias. The distinction between direct GDM evidence, extrapolated mechanisms, and exploratory findings in Table 1 improves transparency but should not be interpreted as a substitute for formal quality appraisal. The proposed clinical framework should consequently be regarded as an approach requiring validation.

### 8.3. Prioritized Research Directions

Research priorities should be ordered according to feasibility and the questions they can resolve. An immediate, relatively tractable priority is harmonization of working definitions, laboratory methods, and reporting. Studies should distinguish isolated autoantibody positivity from persistent or multiple autoantibodies accompanied by impaired β-cell function. Reporting should specify assay platforms, positivity thresholds, antibody levels, gestational age at sampling, and the timing of insulin initiation. Harmonization and reanalysis of existing cohorts may help establish comparable phenotypes before more complex predictive approaches are developed.

A second priority, more demanding but central to the stress-test framework, is prospective recruitment before conception or in early pregnancy. Serial measurements of autoantibodies, stimulated C-peptide, and insulin sensitivity, followed through pregnancy and postpartum, could clarify the temporal relationship between autoimmunity and gestational hyperglycemia. Preconception cohorts are particularly informative because they can document abnormalities before pregnancy-related metabolic and immunological changes occur. Appropriately selected nonpregnant comparison groups would strengthen efforts to distinguish metabolic unmasking from modification of the autoimmune process.

A third priority is clinical validation. Prospective multicenter studies should determine whether selective autoantibody testing and assessment of β-cell function improve classification, postpartum surveillance, and clinically meaningful outcomes beyond standard GDM care. Follow-up should distinguish progression to T1D, development of T2D, persistent dysglycemia, and recovery of glucose tolerance. The benefits, burdens, and cost-effectiveness of additional testing should be evaluated before wider implementation.

Finally, genetic scores, immune-cell profiling, circulating microRNAs, metabolomics, proteomics, and machine-learning approaches should be positioned as later-stage exploratory strategies [37,38,39]. Their evaluation requires sufficiently defined phenotypes, adequate numbers of antibody-positive participants, and independent validation. Priority should be given to demonstrating added value beyond clinical features, antibody profiles, and β-cell function. International collaboration and standardized prospective registries would support progress across these stages.

## 9. Conclusions

GDM includes different biological phenotypes in which insulin resistance and impaired insulin secretion contribute to varying degrees. In some women, islet autoantibodies are accompanied by reduced β-cell reserve and an increased risk of subsequent autoimmune diabetes. Pregnancy may act as a physiological metabolic stress test that reveals previously compensated β-cell dysfunction. However, the timing of autoimmune onset and the influence of pregnancy on its progression remain uncertain. Mechanisms described in T1D offer a possible explanation, but most require direct confirmation in women with GDM.

Autoantibody positivity alone should not be equated with a distinct form of GDM. The strongest evidence of clinically meaningful autoimmune β-cell dysfunction comes from the convergence of persistent or multiple autoantibodies, reduced endogenous insulin secretion and a compatible postpartum course. Universal autoantibody screening, antibody-specific glycemic targets and immune-directed treatment are not supported by current evidence, whereas selective evaluation is reasonable when the phenotype suggests insulin deficiency.

Precision-medicine tools may eventually allow classification based on mechanisms rather than glycemic thresholds alone, but they require extensive validation. Standardized laboratory protocols and large multicenter prospective studies will be essential to establish whether phenotype-based diagnosis and follow-up improve maternal and long-term metabolic outcomes.

## Figures and Tables

**Table 1:** Evidence assessment and interpretation.

Domain	Evidence Level in GDM	Interpretation
Enrichment of islet autoantibodies	Direct; multiple cohorts and pooled analysis	Established association; frequency varies by population and assay
Postpartum progression to autoimmune diabetes	Direct, longitudinal	Strongest with persistent or multiple antibodies; absolute risk varies
Reduced β-cell reserve, greater insulin requirement	Direct but heterogeneous, observational	Supports impaired secretion in a subset; not diagnostic alone
HLA class II susceptibility	Direct but limited	Consistent with T1D; not recommended for routine classification
Autoreactive CD4^+^/CD8^+^ T-cell destruction	Strong in T1D; sparse in GDM	Plausible extrapolation, not a demonstrated GDM-specific pathway
Cytokine-mediated injury and β-cell stress	Strong experimental/T1D evidence; limited in GDM	Requires pregnancy-specific validation
Th17/Treg imbalance	Evidence in T1D; insufficient in GDM	Hypothesis-generating
Viral triggers, microbiome, vitamin D, environment	Indirect and heterogeneous	Possible modifiers, not established causes
MicroRNA, metabolomic, proteomic, extracellular-vesicle signatures	Studied mainly in unselected GDM	Exploratory; no validated application to the autoimmune phenotype

**Table 2:** Autoimmune spectrum in women with GDM.

Term	Working Interpretation	Important Limitation
Islet-autoantibody-positive GDM	GDM with at least one diabetes-associated islet autoantibody detected during pregnancy	Seropositivity alone does not establish the rate of β-cell loss, permanence of autoimmunity or an independent disease entity
Autoimmune phenotype of GDM	Autoimmunity considered clinically relevant because antibody findings are accompanied by reduced β-cell reserve or a compatible clinical course	No universally accepted diagnostic criteria or validated thresholds
Pregnancy-unmasked autoimmune diabetes	Autoimmune β-cell disease probably present before conception that became clinically apparent because pregnancy increased insulin demand	Overlaps with adult-onset T1D and slowly progressive autoimmune diabetes; classification unsettled
Predominantly insulin-resistant GDM	Hyperglycemia dominated by pregnancy-induced insulin resistance with relative β-cell failure	Autoimmunity and insulin resistance can coexist; not a mutually exclusive category

**Table 3:** Clinical differences between autoimmune and insulin-resistant GDM.

Characteristic	Predominantly Insulin-Resistant GDM	Autoimmune Phenotype of GDM
BMI	Higher	Lower (obesity does not exclude it)
Primary mechanism	Insulin resistance	Reduced β-cell reserve with islet autoimmunity
Insulin sensitivity	Reduced	Often relatively preserved
β-cell reserve	Mildly impaired	Markedly reduced
Islet autoantibodies	Usually negative	Positive; number and persistence matter
C-peptide	Normal/high	Low or declining
Insulin treatment	Variable	More frequent, often earlier
Other autoimmune diseases	Uncommon	May coexist
Long-term outcome	Mainly type 2 diabetes	Risk of T1D or slowly progressive autoimmune diabetes, as well as type 2 diabetes

Note: These features represent general clinical tendencies rather than established diagnostic criteria. C-peptide concentrations during pregnancy should be interpreted in the context of gestational age, concurrent glucose concentration, insulin resistance and sampling conditions, including whether the measurement was fasting or stimulated. The qualitative categories shown here do not represent validated gestational-age-specific diagnostic thresholds.

**Table 4:** Genetic susceptibility and proposed environmental factors potentially relevant to islet autoimmunity in GDM.

Risk Factor	Representative Factors	Potential Mechanism
HLA class II susceptibility	HLA-DR3-DQ2, HLA-DR4-DQ8	Enhanced β-cell autoantigen presentation
Non-HLA susceptibility genes	PTPN22, CTLA4, IL2RA, IFIH1, SH2B3	Impaired immune regulation and peripheral tolerance
Family history of autoimmunity	T1D, autoimmune thyroid disease, celiac disease	Shared genetic susceptibility
Enteroviral infections	Coxsackievirus B, other enteroviruses	Molecular mimicry and bystander activation
Gut microbiota dysbiosis	Reduced microbial diversity	Altered immune homeostasis and Treg dysfunction
Vitamin D deficiency	Low serum 25(OH)D	Reduced immunomodulatory activity
Environmental exposures	Diet, smoking, pollutants	Chronic inflammation and oxidative stress

Note: Most mechanisms listed in this table derive from T1D or experimental research and have not been demonstrated directly in islet-autoantibody-positive GDM. HLA associations have been reported in women with GDM [30], but the listed factors should not be interpreted as established causes of the autoimmune phenotype.

**Table 5:** Biomarkers used for characterization of the autoimmune phenotype of GDM.

Biomarker	Target	Clinical Significance
GADA	GAD65	Most frequent and practical marker of islet autoimmunity in adults
IA-2A	IA-2	Adds specificity; associated with more advanced autoimmunity
ZnT8A	Zinc transporter 8	Increases sensitivity when combined with GADA
ICA	Islet cytoplasmic antigens	Classical marker of islet autoimmunity
IAA	Endogenous insulin	Early autoimmune response; uninterpretable after insulin therapy
C-peptide	Endogenous insulin secretion	Estimates residual β-cell function
Disposition index/insulinogenic index	β-cell function during OGTT	Evaluates insulin secretory capacity

**Table 6:** Potential biomarkers for characterization of the autoimmune phenotype of GDM.

Biomarker	Biological Relevance	Potential Clinical Utility	Current Status
C-peptide	Residual β-cell function	Assessment of β-cell reserve	Available; pregnancy thresholds lacking
Disposition index	β-cell compensation during OGTT	Evaluation of insulin secretory function	Research tool
HLA genotyping	Genetic susceptibility	Identification of women at increased autoimmune risk	Research tool
Polygenic risk scores	Cumulative genetic susceptibility	Individualized risk prediction	Not validated in pregnancy
Immune-cell profiling	Cellular immune activation	Characterization of immune dysregulation	Exploratory
Circulating cytokines	Systemic inflammatory activity	Evaluation of inflammatory status	Non-specific
MicroRNAs	Post-transcriptional gene regulation	Candidate predictive biomarker	Exploratory
Metabolomics	Metabolic alterations	Identification of metabolic signatures	Exploratory
Proteomics	Protein expression patterns	Discovery of disease-associated proteins	Exploratory
Extracellular vesicles	Intercellular communication	Novel circulating biomarkers	Exploratory

**Table 7:** Clinical features suggesting the autoimmune phenotype of GDM.

Clinical Feature	Potential Implication
Lower pre-pregnancy BMI	Less typical of classical insulin-resistant GDM
Early insulin requirement	Reduced endogenous β-cell reserve
Family history of T1D	Genetic predisposition to autoimmunity
Personal history of autoimmune disease	Increased likelihood of autoimmune diabetes
Persistent hyperglycemia despite appropriate therapy	Progressive β-cell dysfunction
Reduced fasting or stimulated C-peptide	Impaired endogenous insulin secretion
Positive thyroid autoantibodies	Autoimmune predisposition

These features should not be interpreted as diagnostic criteria but as indicators of an increased likelihood of autoimmune β-cell dysfunction; clinical judgment remains essential. Differential diagnosis is particularly important when hyperglycemia is severe or atypical. Overt diabetes first recognized in pregnancy, T1D, slowly progressive autoimmune diabetes, monogenic diabetes and secondary diabetes may initially be labeled as GDM if evaluation stops at glucose thresholds [1,21,40,41]. Ketosis, catabolic symptoms, overt-range hyperglycemia, very low C-peptide or persistent postpartum hyperglycemia should prompt urgent reassessment of the diagnosis.

**Table 8:** Clinical interpretation of islet autoantibody in GDM.

Scenario	Reasonable Interpretation	Suggested Action
Single low-titer antibody, preserved β-cell function, no atypical features	Autoimmune signal of uncertain persistence or significance	Confirm result; avoid deterministic labeling; standard GDM management and postpartum testing
Persistent GADA or another islet antibody with reduced or declining C-peptide	More convincing evidence of autoimmune β-cell dysfunction	Endocrinology review; anticipate insulin deficiency; structured postpartum surveillance
Two or more persistent islet autoantibodies	Higher-risk autoimmune phenotype	Specialist follow-up and individualized surveillance; explain that pregnancy-specific absolute risks remain uncertain
Ketosis, catabolic symptoms or overt-range hyperglycemia	Possible T1D or overt diabetes rather than GDM	Urgent reclassification and treatment according to diabetes-in-pregnancy standards
Antibody-negative but low β-cell reserve or atypical phenotype	Autoimmune diabetes possible; alternative causes should be considered	Reassess classification, including monogenic diabetes when appropriate

## Data Availability

No new data were created or analyzed in this study. Data sharing is not applicable to this article.

## Abbreviations

The following abbreviations are used in this manuscript:

GDM	Gestational diabetes mellitus
T1D	Type 1 diabetes
T2D	Type 2 diabetes
GADA	Glutamic acid decarboxylase autoantibodies
GAD65	Glutamic acid decarboxylase 65
IA-2A	Insulinoma-associated antigen-2 autoantibodies
ZnT8A	Zinc transporter 8 autoantibodies
CA	Islet cell antibodies
IAA	Insulin autoantibodies
HLA	Human leukocyte antigen
OGTT	Oral glucose tolerance test
BMI	Body mass index
Th17	T helper 17 cells
Treg	Regulatory T cells
IL-17	Interleukin-17
